# Results from a real-time dosimetry study during left atrial ablations performed with ultra-low dose radiation settings

**DOI:** 10.1007/s00399-021-00762-7

**Published:** 2021-05-11

**Authors:** T. Schreiber, N. Kähler, S. Biewener, V. Tscholl, P. Nagel, P. Attanasio, U. Landmesser, M. Huemer

**Affiliations:** Department of Cardiology, Charité – Universitätsmedizin Berlin, Campus Benjamin Franklin, Hindenburgdamm 3, 12203 Berlin, Germany

**Keywords:** Dosimetry, Ablation, Radiation dose, Frame-rate reduction, Radiation protection, Dosimetrie, Ablation, Strahlendosis, Reduktion der Bildrate, Strahlenschutz

## Abstract

**Background:**

Three-dimensional mapping systems and the use of ultra-low dose radiation protocols have supported minimization of radiation dose during left atrial ablation procedures. By using optimal shielding, scattered radiation reaching the operator can be further reduced. This prospective study was designed to determine the remaining operator radiation exposure during left atrial catheter ablations using real-time dosimetry.

**Methods:**

Radiation dose was recorded using real-time digital dosimetry badges outside the lead apron during 201 consecutive left atrial fibrillation ablation procedures. All procedures were performed using the same X‑ray system (Siemens Healthineers Artis dBc; Siemens Healthcare AG, Erlangen, Germany) programmed with ultra-low dose radiation settings including a low frame rate (two frames per second), maximum copper filtration, and an optimized detector dose. To reduce scattered radiation to the operators, table-suspended lead curtains, ceiling-suspended leaded plastic shields, and radiation-absorbing shields on the patient were positioned in an overlapping configuration.

**Results:**

The 201 procedures included 139 (69%) pulmonary vein isolations (PVI) (20 cryoballoon ablations, 119 radiofrequency ablations, with 35 cases receiving additional ablation of the cavotricuspid isthmus) and 62 (31%) PVI plus further left atrial substrate ablation.

Mean radiation dose measured as dose area product for all procedures was 128.09 ± 187.87 cGy ∙ cm^2^ with a mean fluoroscopy duration of 9.4 ± 8.7 min. Real-time dosimetry showed very low average operator doses of 0.52 ± 0.10 µSv. A subanalysis of 51 (25%) procedures showed that the radiation burden for the operator was highest during pulmonary vein angiography.

**Conclusion:**

The use of ultra-low dose radiation protocols in combination with optimized shielding results in extremely low scattered radiation reaching the operator.

## Abstract (de)

**Hintergrund:**

Der Einsatz von dreidimensionalen Mapping-Systemen und von Niedrigdosiseinstellungen der Röntgenanlage führte zu einer Minimierung der Strahlenbelastung bei linksatrialen Ablationen. Optimierte Abschirmung kann die Streustrahlung als Strahlenbelastung des Untersuchers weiter reduzieren. In dieser prospektiven Studie wurde untersucht, welcher Strahlenbelastung der Untersucher unter Anwendung dieser Maßnahmen während linksatrialer Ablationen noch ausgesetzt ist.

**Methoden:**

Die Strahlenbelastung wurde mittels Echtzeitdosimetrie an der Außenseite der Bleischürze während 201 konsekutiven linksatrialen Ablationen gemessen. Alle Prozeduren wurden mit demselben Röntgensystem (Siemens Healthineers Artis dBc; Siemens Healthcare AG, Erlangen, Deutschland) und mit strahlensparenden Einstellungen durchgeführt, unter anderem mit einer niedrigen Bildrate von 2 Bildern/s, maximaler Kupferfilterung und angepasster Detektoreingangsdosis. Um Streustrahlung zu reduzieren, wurden die Seitenlamellen, die mobile Acrylscheibe und die strahlenabsorbierenden Schilde auf dem Patienten überlappend angeordnet.

**Ergebnisse:**

Die 201 Prozeduren umfassten 139 (69 %) Pulmonalvenenisolationen (PVI; 20 Kryoballonablationen, 119 Radiofrequenzablationen, in 35 Fällen mit zusätzlicher Ablation des kavotrikuspidalen Isthmus) und 62 (31 %) PVI mit zusätzlicher linksatrialer Substratmodifikation. Die Strahlendosis als Dosis-Flächen-Produkt (DAP) betrug durchschnittlich 128,09 ± 187,87 cGy ∙ cm^2^ bei einer Fluoroskopiedauer von im Mittel 9,4 ± 8,7 min. Die per Echtzeitdosimetrie erhobene mittlere Strahlendosis des Untersuchers zeigte sich mit 0,52 ± 0,10 µSv als sehr gering. Eine Subanalyse bei 51 (25 %) Prozeduren zeigte, dass die Strahlendosis des Untersuchers während der Pulmonalvenenangiographie am höchsten war.

**Schlussfolgerung:**

Die Kombination von Niedrigdosiseinstellungen und optimierter Abschirmung führt zu einer extrem niedrigen Streustrahlung als Strahlenbelastung des Untersuchers.

## Introduction and background

Despite the widespread use of non-fluoroscopic catheter visualization, fluoroscopy remains an essential part of catheter and wire localization. One of the drawbacks of the ever-increasing number of complex and prolonged ablation procedures is the higher radiation burden to the patient and the operator; it is well documented that even low-dose radiation can lead to potentially fatal radiation damage [13].

Increased awareness lead to the development of ultra-low dose programs with optimized image processing and system settings, resulting in average doses that equal as little as 1% of an ablation procedure performed 6 years ago [2, 6].

Most dose reduction studies [2, 3, 5, 9, 19] used the dose area product (DAP) or some variation thereof as primary endpoint, which mainly serves to measure the patient’s radiation exposure [13]. However, the DAP cannot precisely determine the radiation burden of the operator, since it depends on factors like shielding, angulation, and distance to the X‑ray system.

Therefore, the purpose of this study was to determine the amount of radiation received by the operator as measured by dosimeters during atrial fibrillation ablation procedures.

The authors hypothesized that by using overlapping shielding and an up-to-date ultra-low-dose protocol, scattered radiation reaching the operator would be vastly reduced.

They also sought to determine the moments during the ablation procedure in which radiation exposure to the operator is particularly high.

## Methods

### Study design

This study is an observational single-center study including 201 patients that underwent left atrial catheter ablation (radiofrequency or cryoballon) due to atrial fibrillation. All patients were consecutively enrolled at the Charité University Hospital, Campus Benjamin Franklin, Berlin, Germany. All patients were treated by the same two senior electrophysiologists using the same Siemens Artis dBc systems (Siemens Healthcare AG, Erlangen, Germany).

Demographic parameters, including body mass index (BMI), age, gender, and comorbidities were documented. Relevant procedural parameters for this study included total procedure time, fluoroscopy time, total radiation dose (measured as DAP), as well as acute procedural success and major periprocedural complications.

### Standard radiation protection

Emphasis was put on general “as low as reasonably achievable” (ALARA) measurements, which the authors previously described [24]. These included avoiding magnification and unnecessary fluoroscopy/cine loops, maximal collimation, preferring anteroposterior (AP) and right anterior oblique (RAO) views over steep left anterior oblique (LAO) angulation, and minimizing the distance between patient and detector.

**Fig. 1:**
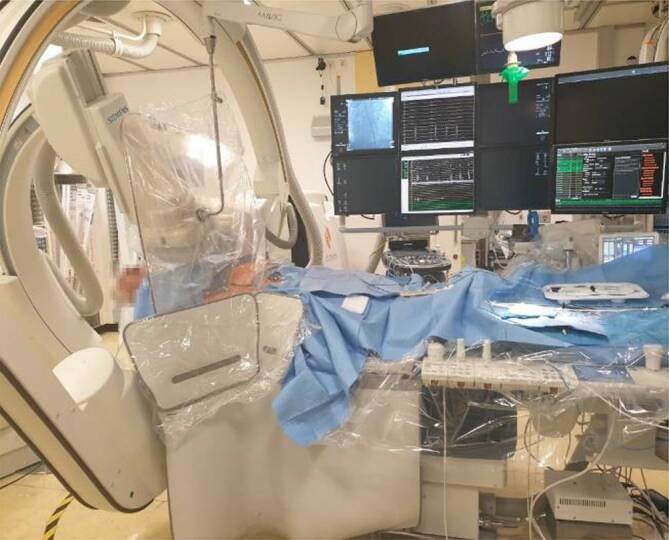
Overlapping shielding with table-suspended lead curtains and ceiling-suspended leaded plastic shields during pulmonary vein isolation

Standard shielding included the use of thyroid and body aprons with 0.5-Pb equivalent. As shown in Fig. 1, table-suspended lead curtains and transparent, ceiling-suspended plastic shields were positioned in an overlapping configuration to ensure maximum protection against scattered radiation. Also, radiation-absorbing shields were placed on the patient.

Furthermore, an ultra-low-dose protocol was uniformly used for all ablation procedures. This consists of the use of a low frame rate (two frames per second, FPS), maximum copper filtration, low detector entrance dose (8 nGy/pulse for fluoroscopy), and short pulse width (4 ms). As previously described, the antiscatter grid was not used in any cases in order to further minimize radiation exposure [1]. For left atrial angiography, short (3-s) cine loops with 7.5 FPS were used during fast left ventricular pacing (200 bpm) via the ablation catheter. The pictures were recorded in biplane mode in RAO 30° or LAO 30° view. No preprocedural imaging was performed.

### Measurement of radiation dose

Two parameters aiming to quantify radiation burden were obtained for each procedure. To evaluate dose to the patient, DAP (cGy ∙ cm^2^) was automatically measured by an ionization chamber. Measurement of DAP is routinely used to compare radiation burden of the patient and correlates well with skin dose [18].

Additionally, electronic dosimetry badges (RaySafe i3, Unfors, Hopkinton, MA, USA) were mounted outside the lead apron at the left side of the chest to measure the operator’s dose (µSv) for each procedure. Both operators were blinded to the measured doses.

This dosimeter is able to measure radiation every second and has high sensitivity (<30 µSv/h). RaySafe dosimeters also have good reproducibility [14] and have already been successfully used in neuroradiological [22], orthopaedic [15, 25], and peripheral endovascular procedures [16].

The RaySafe badges were collected and analyzed every week with DoseViewer software in order to prevent loss of sensitivity. For the last 51 (25%) procedures, radiation burden was analyzed for three different segments of the ablation procedure to assess the sequences with the highest radiation burden.

**Fig. 2:**
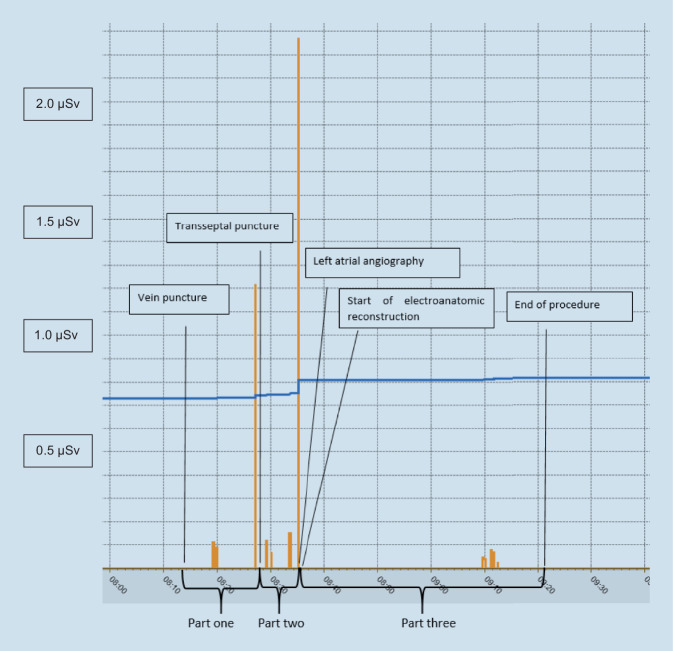
Radiation dispersion during a standard pulmonary vein isolation ablation procedure. The highest peaks can be observed during transseptal puncture and left atrial angiography

The procedures were divided into three parts as follows (see Fig. 2):

- Part one: femoral vein puncture, coronary sinus (CS) catheter placement, transseptal puncture
- Part two: catheter placement, left atrial angiography
- Part three: three-dimensional (3D) mapping, catheter ablation

### Left atrial ablation procedures

After exclusion of intracardiac thrombi by transesophageal echocardiography, pulmonary vein isolation (PVI) or PVI plus further left atrial substrate modification or PVI plus ablation of the cavotricuspid isthmus (CTI) were performed under conscious sedation with midazolam and propofol. A diagnostic 10-poled steerable electrophysiology catheter (Inquiry, St Jude Medical, Saint Paul, MN, USA) was placed in the CS. Double transseptal puncture was performed in all cases and two catheters were introduced via two long left atrial sheaths (Swartz Braided SL0 Curve 8.5 F, St. Jude Medical, Saint Paul, MN, USA, and Agilis *N*xt Small Curl 8.5F, Abbott, MN, USA):

- A circular 12-pole pulmonary vein mapping catheter (Lasso Nav Variable, Biosense Webster, Diamond Bar, CA, USA; via the Swartz sheath)
- An open-irrigated 3.5-mm tip Thermocool Smarttouch SF (Biosense Webster) as ablation catheter (via Agilis)

After electroanatomical reconstruction of the left atrium, catheter ablation was performed guided by ablation index (AI) with irrigated radiofrequency energy at a power of 20–25 W at the posterior left atrial wall (target AI, 400) and 30–35 W at all other sites (target AI, 550).

### Statistical analysis

All analyses were performed using SPSS software version 25 (SPSS Inc., Chicago, IL, USA). Data are presented as mean ± standard deviation (SD) for continuous variables or absolute numbers and percentages for categorical variables. For normally distributed data, correlation values were calculated with Pearson’s correlation coefficient and Spearman for skewed data. A *p*-value of <0.05 was considered statistically significant.

## Results

The 201 procedures included 139 (69%) PVI (20 cryoballoon ablations and 119 radiofrequency ablations with 35 cases receiving additional ablation of the CTI) and 62 (31%) PVI plus further extra pulmonary substrate ablations. Table 1 shows baseline characteristics, Table 2 shows procedural characteristics of the study cohort.

**Table 1:** Baseline characteristics

Female, *n* (%)	86 (43%)
Age, mean (±SD)	68.31 (±10.04)
BMI, mean (±SD) (kg/m^2^)	27.83 (±4.85)
LVEF (±SD) (%)	55.96 (±9.95)
Paroxysmal atrial fibrillation, *n* (%)	85 (42%)
Diabetes mellitus, *n* (%)	33 (16%)
Arterial hypertension, *n* (%)	154 (77%)
COLD, *n* (%)	7 (3%)
CAD, *n* (%)	52 (26%)

*SD* standard deviation, *BMI* body mass index, *LVEF* left ventricular ejection fraction, *COLD* chronic obstructive lung disease, *CAD* coronary artery disease

**Table 2:** Procedural characteristics

DAP, mean (±SD) (µGy ∙ m^2^)	128.09 (±187.87)
Dose to the operator, mean (±SD) (µSv)	0.52 (±0.10)
Procedural duration, mean (±SD) (h:min)	1:37 (±0:30)
Fluoroscopy time, mean (±SD) (min)	9.4 (±8.7)
DAP per minute, mean (±SD)	14.09 (±15.77)
Acute procedural success, *n* (%)	201 (100%)
Complications, *n* (%)	9 (4%)

*DAP* dose area product, *SD* standard deviation

Mean radiation dose measured as DAP for all procedures was 128.09 + 187.87 cGy ∙ cm^2^ with a mean fluoroscopy duration of 9.4 + 8.7 min. The 10 highest recorded doses predominantly (90%) occurred during ablation procedures of patients with a BMI >30. We observed a significant positive relationship between the patients BMI and operator dose/DAP (R = 0.348, *p* < 0.001 and R = 0.506, *p* < 0.001).

**Fig. 3:**
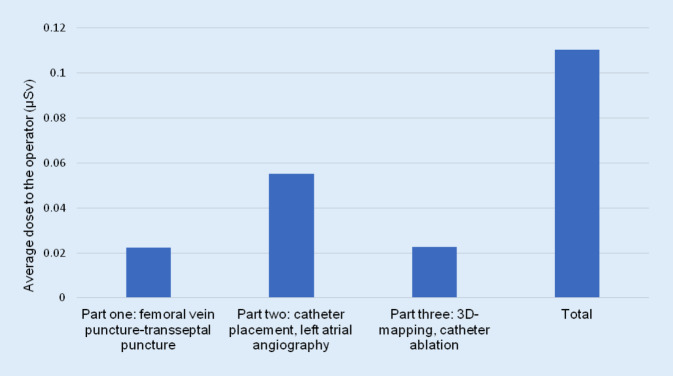
Distribution of radiation throughout the different parts of the ablation procedure

As mentioned earlier, for the last 51 procedures, a subanalysis was performed to determine which part of the procedure showed the highest radiation exposure to the operator. Figure 3 shows that most radiation (55%) towards the operator occurs during left atrial angiography.

## Discussion

### Main findings

This single-center study demonstrated that, with the use of an ultra-low-dose program and optimized shielding, operator doses can be kept at very low levels during catheter ablation of atrial fibrillation. The operator’s exposure was highest during left atrial angiography, making shielding and further dose reduction particularly valuable assets at this moment in the procedure. To our knowledge, this is the first analysis of the operator’s exposure with the use of ultra-low-dose programs for catheter ablation of atrial fibrillation.

### Previously published studies

Scant data exist on personal dosimetry regarding atrial fibrillation ablation. A 2019 study reported mean values of 3 µSv with a mean DAP of 429.6 cGy ∙ cm^2^ [10]. Those results were achieved by using a frame rate of 4 FPS. By using a lower frame rate of 2 FPS, the authors were able to achieve lower average doses while maintaining a low rate of complications with a reasonable procedural time. Reducing the frame rate is arguably one of the simplest and most effective ways to lower radiation burden, given that the relationship between dose and frame rate is linear. Despite this fact, only 50% of European ablation centers use a frame rate of less than 6 FPS for electrophysiology (EP) procedures [9]. The reason for this might be that a considerable number of EP labs use settings also used in coronary interventions. However, the demand for image quality in EP studies is usually far more modest, even in complex procedures. Therefore, the authors advise contacting the X‑ray system’s manufacturer to establish a low-dose radiation setup tailored to the operators’ requirements.

Since scattered radiation emitted from the patient’s body is the main source of the operator’s radiation burden, methods to absorb scattered radiation have been tested: sterile radiation-absorbing drapes placed around the puncture site can reduce the operator’s radiation exposure by more than half [12]. Mobile full-body protection systems can provide protection against scattered radiation, except for the arms, and can reduce radiation to the operator close to background radiation values [8]. High costs, limited availability, and structural preconditions have hindered their widespread use.

### Radiation put into context

**Fig. 4:**
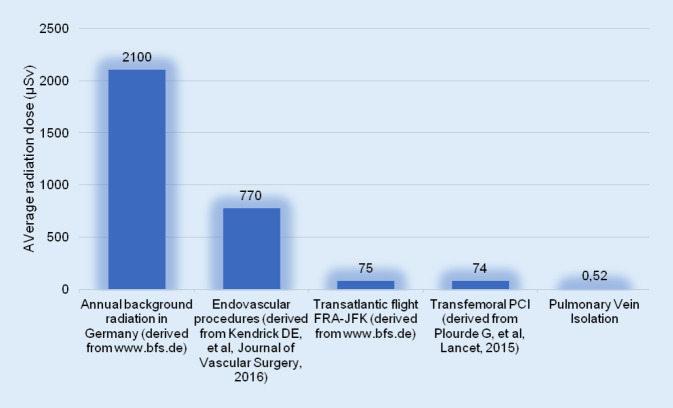
Comparison between annual background radiation and different interventions. *PCI* percutaneous coronary intervention, *FRA-JFK* Frankfurt-John F. Kennedy

Despite various efforts to minimize operator radiation, interventional cardiologists in most centers are still subjected to a relevant amount of radiation throughout their career, with an annual estimated dose of 5 mSv [13]. Reducing radiation burden minimizes the detrimental effects of ionizing radiation, some of which include cancer, inherited diseases, and the formation of cataracts [21]. Figure 4 shows previously published radiation exposure for different diagnostic and therapeutic interventions, as well annual background radiation and radiation during an intercontinental flight.

### Minimizing radiation during left atrial angiography

Knowledge of pulmonary vein anatomy and its variations (common ostia, accessory pulmonary vein, pulmonary vein fusion) is a key factor for a safe and efficient ablation procedure. This information is most frequently obtained by intraprocedural angiography. Preprocedural computed tomograhy left atrial angiography might be helpful to avoid intraprocedural angiography and thereby reduce operator X‑ray exposure. However, this method is associated with a higher radiation burden to the patient [4, 17]. The authors suggest implementing the following measures to ensure low radiation burden:

- Using short cine loops with low frame rates (for example, 3 s and 7.5 FPS) with fast left ventricular pacing to lower use of contrast agent [23]
- Strict collimation to the left atrium
- Avoiding additional angiography of single pulmonary veins

The feasibility and safety of numerous fluoroless approaches for catheter-based pulmonary vein ablation and LA visualization have been demonstrated in smaller studies [7]. These include magnetic resonance imaging-guided ablation procedures [26], as well as intracardiac echocardiography [11] and transesophageal echocardiography [20] without any subsequent LA angiography. However, limited experience and availability, high costs, and possible complications have hindered more widespread use.

## Limitations

This study represents a single-center analysis with two senior electrophysiologists performing all procedures. Therefore, the observed results may not be valid for other ablation centers with different dose settings. Although standardized shielding was aimed for, different placement of the shields may have altered the results.

## Conclusion

This study demonstrates that by using an ultra-low-dose setting and optimal shielding, radiation reaching the operator can be largely reduced. Therefore, table-suspended lead curtains and ceiling-suspended shields should be positioned in an overlapping configuration to ensure maximum protection against scattered radiation, especially during left atrial angiography.

## Declarations

For this article no studies with human participants or animals were performed by any of the authors. All studies mentioned were in accordance with the ethical standards indicated in each case.
